# The transition from evolutionary stability to branching: A catastrophic evolutionary shift

**DOI:** 10.1038/srep26310

**Published:** 2016-05-24

**Authors:** Fabio Dercole, Fabio Della Rossa, Pietro Landi

**Affiliations:** 1 Department of Electronics, Information, and Bioengineering, Politecnico di Milano, Milano, Italy; 2 Department of Mathematical Sciences, Stellenbosch University, Stellenbosch, South Africa; 3 Evolution and Ecology Program, International Institute for Applied Systems Analysis, Laxenburg, Austria

## Abstract

Evolutionary branching—resident-mutant coexistence under disruptive selection—is one of the main contributions of Adaptive Dynamics (AD), the mathematical framework introduced by S.A.H. Geritz, J.A.J. Metz, and coauthors to model the long-term evolution of coevolving multi-species communities. It has been shown to be the basic mechanism for sympatric and parapatric speciation, despite the essential asexual nature of AD. After 20 years from its introduction, we unfold the transition from evolutionary stability (ESS) to branching, along with gradual change in environmental, control, or exploitation parameters. The transition is a catastrophic evolutionary shift, the branching dynamics driving the system to a nonlocal evolutionary attractor that is viable before the transition, but unreachable from the ESS. Weak evolutionary stability hence qualifies as an early-warning signal for branching and a testable measure of the community’s resilience against biodiversity. We clarify a controversial theoretical question about the smoothness of the mutant invasion fitness at incipient branching. While a supposed nonsmoothness at third order long prevented the analysis of the ESS-branching transition, we argue that smoothness is generally expected and derive a local canonical model in terms of the geometry of the invasion fitness before branching. Any generic AD model undergoing the transition qualitatively behaves like our canonical model.

Twenty years ago, S.A.H. Geritz, J.A.J. Metz, and coauthors^1^,^2^,^3^ introduced a mathematical framework—referred to as *Adaptive Dynamics* (AD)—for modeling long-term evolutionary dynamics, with special emphasis on the generation of diversity through *evolutionary branching*. AD has been successfully developed and applied in and outside biology (see ref. 4 and refs therein) as well as debated, mainly due to its asexual nature (see vol. 18 of the *J. Evol. Bio.*).

Evolutionary branching takes off when a resident and a similar mutant type coexist in the same environment and natural selection is disruptive, i.e., favors outer rather than intermediate phenotypes. In the restricted (but classical) formulation in which resident individuals are characterized by the same value *x* of a one-dimensional strategy, Geritz, Metz *et al.*^1^,^2^,^3^ derived explicit conditions for evolutionary branching in terms of the *invasion fitness*^5^—the exponential rate of growth *s*_*x*_(*y*) initially shown by a mutant strategy *y* appeared when the resident is at its ecological regime.

Subsequently, analogous conditions have been derived for models including sexual characters^6^,^7^, spatial distribution^8^, and multi-dimensional phenotypes^9^, establishing evolutionary branching as the prerequisite step for *sympatric* and *parapatric* speciation^10^.

Evolutionary branching requires, first, the resident strategy *x* to be in the vicinity of a convergence stable singular strategy x*—a strategy making neutral the selection pressure measured by the fitness gradient ∂_*y*_*s*_*x*_(*y*)|_*y*=*x*_, i.e.,


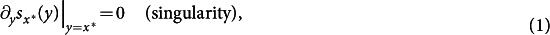


and locally attracting the evolutionary dynamics driven by rare and small strategy mutations^1^,^2^,^3^,^11^,^12^ (convergence stability).

Second, there should be pairs (*x*_1_, *x*_2_) close to the singular point (x*, x*) for which a resident *x* = *x*_1_ and a mutant *y* = *x*_2_ can coexist. For this, a sufficient condition is a negative fitness cross-derivative, i.e.,


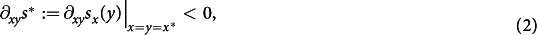


that implies the existence, locally to (x*, x*), of a conical region of coexistence in the strategy plane (Fig. 1). Geritz, Metz *et al.*^1^,^2^,^3^ showed that mutual invasibility occurs in the region, i.e., that (2) implies the invasion fitness of strategy *x*_2_ against the resident *x*_1_, 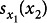, and vice versa, 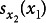, to be both positive (and vanishing each on one of the region boundaries). The existence of a unique ecological attractor of coexistence was shown more recently^13^,^14^,^15^ for the class of unstructured ecological models—no individual distinction w.r.t. age, size, location, etc.—under stationary coexistence. (See Fig. 1c, where 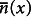 is the equilibrium density of the resident before mutant invasion and 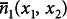 and 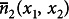 are the equilibrium densities of coexistence).

Third, selection must be disruptive, a sufficient condition for which being


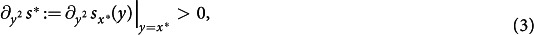


i.e., the opposite of the *evolutionary stability* of the singular strategy^16^,^17^ (see Fig. 1e,f).

Condition (3) was obtained^1^,^2^,^3^ based on the twice differentiability of the *dimorphic fitness* 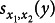
—the invasion fitness of a mutant *y* invading an environment set by the two residents *x*_1_ and *x*_2_ at their ecological regime—and by expanding it around (*x*_1_, *x*_2_, *y*) = (x*, x*, x*) (see Appx. 1 in ref. 3 for details). Exploiting the consistency relations

C1: 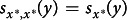, the link between the dimorphic and monomorphic fitness functions at the singular point,

C2: 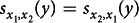, the order irrelevance of the two residents, and

C3: 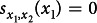 (a) and 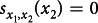 (b), the selective neutrality of the two residents,

Geritz, Metz *et al.*^1^,^2^,^3^ arrived to the following expansion


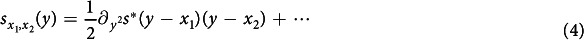


It says that the selection pressures on strategies *x*_1_ and *x*_2_, as measured by the fitness gradients


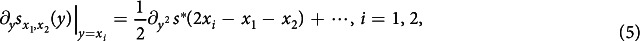


are opposite for (*x*_1_, *x*_2_) in the region of coexistence locally to (x*, x*), meaning that dimorphic evolution points away or toward (x*, x*) if 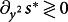 (see Fig. 1f,e, respectively).

The smoothness of the dimorphic fitness 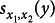 at the singular point (*x*_1_, *x*_2_) = (x*, x*) is a controversial open problem of AD. In ref. 1 (Sect. 6.3.2), the twice differentiability is shown geometrically (see Methods for an algebraic proof) and the authors argue that higher-order derivatives may fail to exist at the singular point. The source of the expected nonsmoothness is the ecological attractor of coexistence being undefined when *x*_1_ = *x*_2_. Think, e.g., of the equilibrium densities 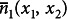 and 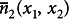 in Fig. 1. They are evidently discontinuous at (x*, x*), respectively being equal to zero and 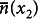 along the extinction boundary 1 (blue) and to 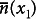 and zero along the extinction boundary 2 (red). The nonsmoothness of the dimorphic fitness arising at 3^rd^ order remained a mystery of AD that long prevented the analysis of the ESS-branching transition^18^.

The nonsmoothness conjecture was recently disproved (exploiting a new structural ecological property^19^) for the class of unstructured ecological models under stationary coexistence. We have indeed shown^20^ the smoothness of the dimorphic fitness up to 3^rd^ order. Moreover, our methodology is general up to any order and there is thus no reason to expect nonsmoothness to arise at some higher order.

Here we lift the above result to the generality of any class of ecological models, based on the newly discovered smoothness of the dimorphic fitness. Only assuming a three-times differentiable dimorphic fitness, we show that the consistency relations C1–C3 (with C1 considered along the extinction boundaries delimiting the coexistence region, see Methods) determine the expansion of the dimorphic fitness up to 3^rd^ order, and the result is the same as obtained in ref. 20. That is, the 1^st^ order is null, the 2^nd^ coincides with (4), and the 3^rd^ is


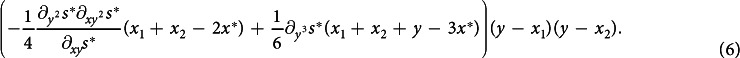


Note that the expansion is given in terms of the monomorphic fitness derivatives (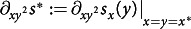 and 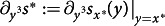 are the involved third derivatives)—that are determined by the ecological dynamics before branching^5^—in contrast to what preliminarily expected based on the analysis of Lotka-Volterra models^21^. Unfortunately, the consistency arguments offered by relations C1–C3 do not determine the fitness expansion at higher orders, so whether the local approximation of the dimorphic fitness is fully controlled by the geometry of the monomorphic fitness remains an open question. Any answer necessarily requires specifying the class of underlying ecological models, so that one can proceed with the direct computation of the fitness expansion, as in ref. 20.

Our analysis allows to go beyond 2^nd^ order in the analysis of the branching dynamics, thus solving the 20-yrs-lasting impasse. In particular, the analysis at 3^rd^ order is of fundamental importance, because necessary to unfold the mechanisms underlying the ESS-branching transition (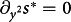 under the coexistence condition (2), see Fig. 2), close to which the branching dynamics is dominated by the 3^rd^-order terms in the approximation of the dimorphic fitness. Note that close to the transition 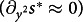 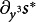 is the leading 3^rd^-order coefficient in (6), so we perform our analysis assuming the genericity condition


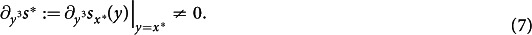


The analyses at 4^th^ and higher orders will be possible only under specific ecological assumptions and are interesting for the understanding of degenerate branching scenarios^22^, where some of the fitness derivatives vanish at the singularity due to model-specific properties, like symmetries in the phenotypic dependence of demographic parameters around optimal values.

Based on the 3^rd^-order expansion (4, 6), we derive the following canonical model for the ESS-branching transition:


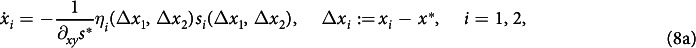



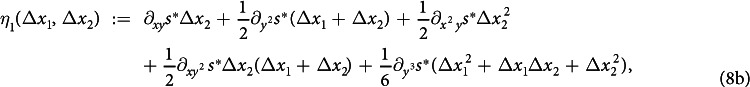



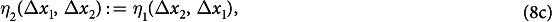



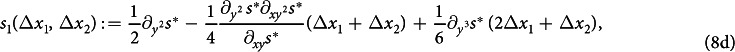



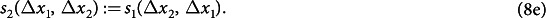


The evolutionary dynamics of model (8) are depicted (under 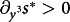) in Fig. 2 in the coexistence region delimited by the extinction boundaries *η*_1_(Δ*x*_1_, Δ*x*_2_) = 0 (blue) and *η*_2_(Δ*x*_1_, Δ*x*_2_) = 0 (red). Within the coexistence region, 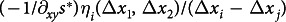 represents the birth output of populations *i* at the ecological regime and *s*_*i*_(Δ*x*_1_, Δ*x*_2_)(Δ*x*_i_ − Δ*x*_*j*_), *i* ≠ *j*, approximates the fitness gradient (5) (see Methods). The (black) trajectories describe the continuous dynamics obtained in the limit of infinitesimally small mutational steps^11^,^12^ (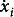 standing for the time-derivative of *x*_*i*_ on a suitable evolutionary time scale), but the fitness approximation and the direction of dimorphic evolution describe as well the evolution driven by finitely small mutations^23^,^24^. The dashed (blue and red) lines solve *s*_1_(Δ*x*_1_, Δ*x*_2_) = 0 and *s*_2_(Δ*x*_1_, Δ*x*_2_) = 0 and therefore approximate the (*x*_1_ and *x*_2_) *nullclines* of the evolutionary dynamics (the lines on which selection is neutral in strategy *x*_1_ and *x*_2_, respectively, so that evolution points vertically and horizontally). The nullclines and the extinction boundaries (the latter also behaving as nullclines with same color code) are known to connect at special points^1^,^25^—the boundary equilibria with one strategy at the singularity x*, the other being absent (connections with different colors, half-filled points), and the points where the absent strategy is (potentially) singular (same color connections, occurring at the horizontal and vertical extremal points of the extinction boundaries). Model (8) shows such connections while approaching the ESS-branching transition.

The unfolding of Fig. 2 has been observed in previous analyses of specific models^24^,^25^,^26^,^27^ and conjectured to be general based on the above described properties of the evolutionary nullclines^28^,^29^. Our contribution at last gives the full theoretical support. We have shown that any single or multi-species community undergoing the ESS-branching transition due to gradual changes in, e.g., climate, nutrients, habitat fragmentation, or biotic control and exploitation qualitatively behaves like model (8) near the singular point (x*, x*).

Figure 2 clearly shows the mechanisms underlying the ESS-branching transition. Restricting the attention to the coexistence region above the diagonal (since relation C2 implies the symmetry w.r.t. the diagonal), the boundary equilibria (half-filled) at *x*_1_ = x* (left) and at *x*_2_ = x* (right) behave as saddles for the dimorphic evolutionary dynamics, attracting along one trajectory—the *stable manifold* of the saddle—and repelling for all other nearby initial conditions. In the ESS case (left), the saddle’s stable manifold separates the initial conditions leading to the ESS—a dimorphic phase up to the extinction of one of the two populations followed by the monomorphic convergence (represented on the diagonal) to the ESS—from those leading away, typically to a nonlocal evolutionary attractor not involved in the transition^25^. In the branching case (right), the initial conditions near the singular point (x*, x*) all lead away. Approaching the transition (left-to-right) along with gradual changes in the model parameters, the boundary saddle approaches the ESS, so do the initial conditions leading away. Note that branching is possible at the transition too (for mutant strategy *x*_2_ larger than x*, central panel; branching at the transition symmetrically requires *x*_2_ < x* under 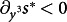, not shown, see Methods).

In real systems, where mutational steps are small but finite^23^,^24^, the monomorphic dynamics converging to the singular strategy x* eventually jump into the dimorphic region. In an ESS case far from the transition (see Fig. 2, left, at a scale at which mutations are sufficiently small), the dimorphic dynamics jump back monomorphic a few steps later, while the distance to the ESS keeps contracting (see ref. 1, Sect. 3.2.4). However, close to the transition, the initial conditions that trigger the branching dynamics become feasible (see again Fig. 2, left, at a scale at which mutations are large enough to reach initial conditions above the saddle’s stable manifold). Branching is therefore possible before the transition actually occurs and is properly discussed in term of finite mutations—since infinitesimal mutations cannot reach the coexistence region away from the singular strategy x* (see Methods for further detail). In the branching case (Fig. 2, right), the dimorphic dynamics eventually lead away.

The new evolutionary attractor reached after branching is already viable before the transition^25^ and this shows that the environment is ready to host populations 1 and 2 before branching becomes feasible. Invasion by alien phenotypes could therefore anticipate the endogenous diversity that branching brings about. Whether the new attractor is dimorphic or evolution steps back monomorphic discriminates long-term from temporary diversity. Repeated branching and extinction^30^,^31^ could also be triggered by the ESS-branching transition, showing a peculiar case in which the new evolutionary attractor is actually involved in the transition.

The ESS-branching transition is irreversible—a *catastrophic evolutionary shift*^32^—in the sense that a small change in a model parameter causes an evolutionary transient toward a nonlocal attractor, and once the transient is triggered it can not be reversed by counteracting the parameter perturbation. Weak evolutionary stability—quantified by a small negative 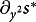 and possibly testable^33^ by estimating the invasion fitness of artificially introduced similar strategies—hence qualifies as an early-warning signal for branching and a measure of the community’s *resilience* against biodiversity.

As a catastrophic evolutionary shift, the ESS-branching transition bridges the meso and macro scales of evolution^28^. The short periods of rapid morphological change observed by paleontologists in the fossil record, along with gradual environmental changes on the macro scale—the so-called *punctuated equilibria*^34^—could correspond to the branching dynamics triggered by the transition and developing on the meso scale.

This work fully explains one of the most important “phase transition” in Nature.

## Methods Summary

We consider two similar populations, with densities *n*_1_ and *n*_2_ and one-dimensional strategies *x*_1_ and *x*_2_ ≈ *x*_1_. In the monomorphic phase populations 1 and 2 are resident and mutant, respectively, whereas they are both residents in the dimorphic phase. We do not specify a particular class of ecological models, but only assume that the resulting monomorphic and dimorphic invasion fitnesses are smooth (only differentiability up to 3^rd^ order is actually required). In particular for the dimorphic fitness 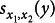, smoothness at (*x*_1_, *x*_2_, *y*) = (x*, x*, x*) means that a polynomial local expansion in 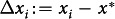, *i* = 1, 2, Δ*y* := *y *− x* holds good in the resident-mutant coexistence region—the error of a truncated expansion of order *k* is 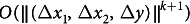 when (Δ*x*_1_, Δ*x*_2_) vanish along a path in the coexistence region.

The above smoothness assumption is currently proved for the class of unstructured ecological models under stationary coexistence^20^, though we expect it to hold with wide generality. Specifically, smoothness is generically expected radially from the singular point (x*, x*) in the strategy plane (*x*_1_, *x*_2_)^1^. Then, showing the smoothness of the dimorphic fitness at (*x*_1_, *x*_2_, *y*) = (x*, x*, x*) reduces to showing that the directional expansion along rays (*x*_1_, *x*_2_) = (x* + *ε* cos *θ*, x* + *ε* sin *θ*) at given *θ* and around *y* = x* is polynomial in the direction components (cos *θ*, sin *θ*). However, without specific assumptions on the underlying ecological model, this is possible only up to order 2 (see SI, Sect. 1).

Using a 3^rd^-order expansion of the dimorphic fitness and imposing the consistency relations C1–C3, with C1 replaced by


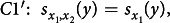


along the extinction boundary 2 on which only strategy *x*_1_ is present, we derive the expansion (4, 6) under the coexistence condition (2) (note that imposing the monomorphic-dimorphic link on the extinction boundary 1 is redundant due to the diagonal symmetry between the two boundaries and property C2, see SI, Sects. 2 and 3).

**Figure 1:**
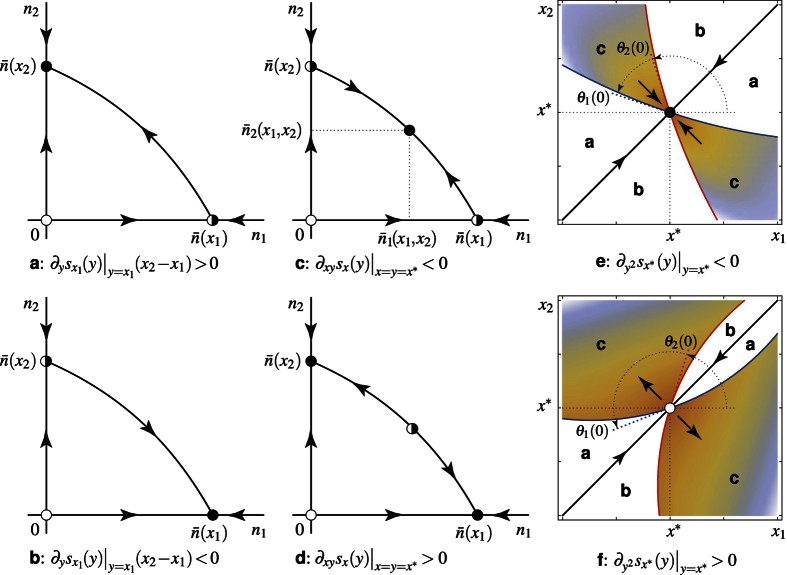
Resident-mutant competition scenarios close to a singular strategy x*. Mutant dominance (**a**) and resident dominance (**b**) occur away from the singularity (*x*_1_ ≠ x*) for *x*_2_ sufficiently close to *x*_1_. Coexistence (**c**) occurs under condition (2) for (*x*_1_, *x*_2_) in the region (shaded in (**e**,**f**)) rooted at the singular point (x*, x*) and delimited by the extinction boundaries 1 (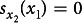 along which 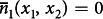, blue) and 2 (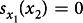 along which 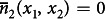, red). Mutual exclusion (**d**) occurs in the region delimited by the boundaries 1 and 2 under the opposite condition (not shown). When coexistence is possible, the singular strategy is an ESS (**e**, mutants with either larger or smaller strategy fail to invade) or a branching point (**f**). The opening angle *θ*_1_(0) − *θ*_2_(0) of the coexistence region is acute in the first and obtuse in the second case. Monomorphic and dimorphic evolutions point in the direction of the black arrows. Full points: attractors; half-filled points: saddles; empty points: repellors.

Specifically, to impose C1′, we use the polar coordinates (*ε*, *θ*) and *ε*-parameterize the extinction boundary 2 as *θ* = *θ*_2_(*ε*), *θ*_2_(*ε*) being the function that gives the angle *θ* of the boundary point at distance *ε* from the singular point (x*, x*). Then, C1′ becomes


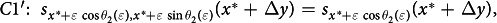


to be imposed together with its (*ε*, Δ*y*)-derivatives at (*ε*, Δ*y*) = (0, 0) up to order 3. This involves the angle *θ*_2_(0)—the tangent direction to the extinction boundary 2 at (x*, x*)—and the first derivative *θ*_2_′(0)—the local curvature of the boundary (whether *θ* increases or decreases while moving away from (x*, x*), see Fig. 1e,f). The two quantities are determined by the second and third derivatives of the monomorphic fitness. They are obtained by imposing the second and third *ε*-derivatives of the boundary definition


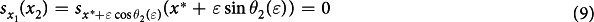


(the first derivative being uninformative).

Unfortunately, the above procedure does not apply at order 4, as the conditions imposed by relations C1′–C3 are less than the number of coefficients in the expansion of the dimorphic fitness. The missing conditions must then come from specific assumptions to be made at the ecological level.

The derivation and the analysis of the canonical model (8) are detailed in the Supplementary Information (Sects. 4 and 5).

## An example

We illustrate the developed body of theory on a well-known example: a single species AD Lotka-Volterra model of asymmetric competition^26^. The resident-mutant ecological model reads:


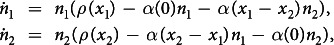


where 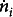 here stands for the time-derivative of *n*_*i*_ on the ecological time scale, *i* = 1, 2, and the strategy *x* scales (from −∞ to +∞) with competitive ability. The intrinsic growth rate *ρ* and the competition function *α* are strategy-dependent, with Gaussian 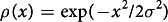, *σ* > 0, that identifies an optimal strategy for a single population, and sigmoidal 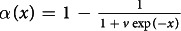, *v* > 0, that gives a competitive advantage to larger strategies.

The model is simple enough that we can solve analytically for all the relevant quantities: the monomorphic and dimorphic resident equilibrium densities (at which 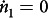 in the absence of population 2 and 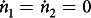 in the presence of both populations)


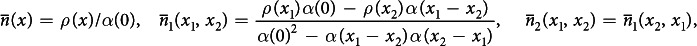


the monomorphic and dimorphic fitnesses


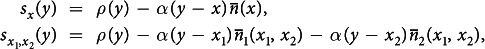


the monomorphic and dimorphic fitness gradients


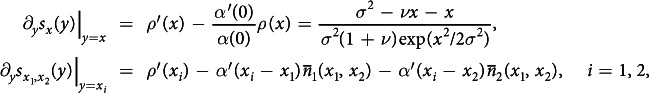


the singular strategy making the monomorphic fitness gradient zero


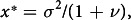


the fitness second derivatives ruling branching at x*


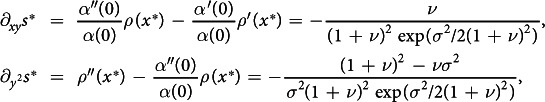


and the third derivatives entering our approximations


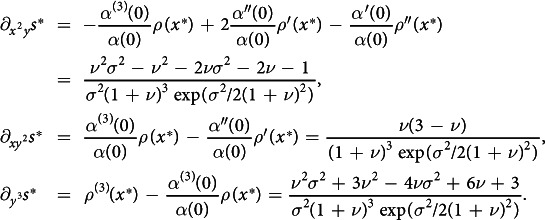


It is easy to verify that the singular strategy x* is attracting the monomorphic evolutionary dynamics for any positive (*σ*, *v*) (the eigenvalue of the continuous dynamics is 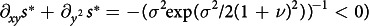, that coexistence in its vicinity is always possible (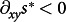 for *v* > 0), and that branching (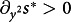) occurs if *σ*^2^ > (1 + *v*)^2^/*v*. At *σ*^2^ = (1 + *v*)^2^/*v* the system undergoes the ESS-branching transition. Increasing the value of *σ*, the ESS turns into a branching point.

**Figure 2:**
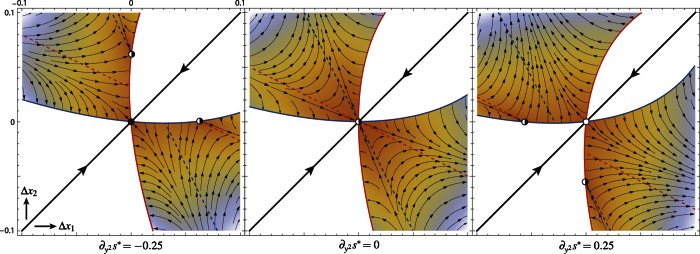
Unfolding of the ESS-branching transition. The flow of model (8) (black trajectories with dashed *x*_1_-blue- and *x*_2_-red-nullclines) is shown in the resident-mutant coexistence region delimited by the extinction boundaries 1 (*η*_1_(Δ*x*_1_, Δ*x*_2_) = 0, see Eq. (8b), blue) and 2 (*η*_2_(Δ*x*_1_, Δ*x*_2_) = 0, see Eq. (8c), red). The panels are symmetric w.r.t. the diagonal Δ*x*_1_ = Δ*x*_2_ (due to property C2). The cubic approximation (4, 6) of the dimorphic fitness gives only a quadratic approximation of the extinction boundaries and a linear approximation of the nullclines w.r.t. their fully nonlinear versions. The approximation of the dimorphic flow is also quadratic in the case of stationary ecological coexistence (see Methods). The figure is obtained for 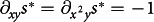, 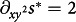, 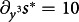. The case with 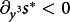 can be obtained through a local symmetry w.r.t. the anti-diagonal Δ*x*_1_ + Δ*x*_2_ = 0.

**Figure 3:**
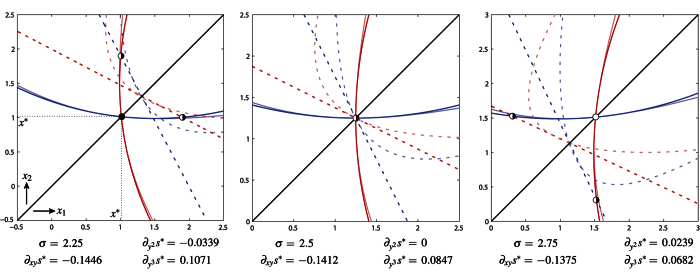
Unfolding of the ESS-branching transition in the AD model in ref.^26^. The model parameter *σ* increases from left to right turning the singular strategy x* from ESS (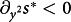) to branching point (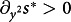) (other parameter: *v* = 4). The approximations *η*_*i*_(Δ*x*_1_, Δ*x*_2_) = 0 and *s*_*i*_(Δ*x*_1_, Δ*x*_2_) = 0 (see model (8)) of the extinction boundaries and of the *x*_*i*_-nullcline, *i* = 1, 2, are shown locally to (x*, x*) using the same graphical and color code of Fig. 2. Lighter colors are used for the fully nonlinear versions: extinction boundary 1, 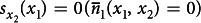; extinction boundary 2, 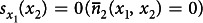; and *x*_*i*_-nullcline, 
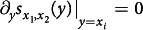
.

Figure 3 compares our (approximating) canonical model (8) and extinction boundaries 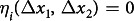, *i* = 1, 2, with their fully nonlinear versions. As in Fig. 2, the extinction boundary 1 and the *x*_1_-nullcline of the dimorphic evolutionary dynamics are plotted in blue (solid and dashed); red for boundary 2 and the *x*_2_-nullcline. Lighter colors are used for the fully nonlinear versions (recall that the approximation is quadratic for the extinction boundaries and linear for the *x*_*i*_-nullclines, locally to the singular point (x*, x*)).

## Additional Information

**How to cite this article**: Dercole, F. *et al.* The transition from evolutionary stability to branching: A catastrophic evolutionary shift. *Sci. Rep.* **6**, 26310; doi: 10.1038/srep26310 (2016).

## Supplementary Material

Supplementary Information
