# Supplementary material for: The transition from evolutionary stability to branching: A catastrophic evolutionary shift
Source: Sci Rep. 2016 May 24;6:26310. doi: 10.1038/srep26310 (PMC4877704; doi:10.1038/srep26310)
Supplement: Supplementary Information [file srep26310-s1.pdf]

Supplementary Information

The transition from evolutionary stability to branching: A catastrophic evolutionary shift

Fabio Dercole[1,*], **Fabio Della Rossa**[1], **and Pietro Landi**[2,3]

[1]Department of Electronics, Information, and Bioengineering, Politecnico di Milano, Milano, Italy
[2]Department of Mathematical Sciences, Stellenbosch University, Stellenbosch, South Africa
[3]Evolution and Ecology Program, International Institute for Applied Systems Analysis, Laxenburg, Austria
[*]fabio.dercole@polimi.it

Methods

1 The twice differentiability of the dimorphic fitness

Let's first be precise on the meaning of differentiability at $(x_1,x_2,y) = (x^*,x^*,x^*)$ for the dimorphic fitness. Since point (x^*,x^*,x^*) is on the boundary of the function's domain—the singular point $(x_1,x_2) = (x^*,x^*)$ being a corner point of the resident-mutant coexistence region—twice differentiability should not be interpreted in the classical sense—existence of first and second partial derivatives of the dimorphic fitness at (x^*,x^*,x^*)—but as the existence of a local polynomial expansion

$$\begin{aligned} s_{x_1,x_2}(y) &= s^* + s^*_{100}\Delta x_1 + s^*_{010}\Delta x_2 + s^*_{001}\Delta y \\ &+ \tfrac{1}{2}s^*_{200}\Delta x_1^2 + s^*_{110}\Delta x_1\Delta x_2 + s^*_{101}\Delta x_1\Delta y + \tfrac{1}{2}s^*_{020}\Delta x_2^2 + s^*_{011}\Delta x_2\Delta y + \tfrac{1}{2}s^*_{002}\Delta y^2 + \cdots, \end{aligned} \tag{M1}$$

$\Delta x_i := x_i - x^*$, $i = 1,2$, $\Delta y := y - x^*$, that guarantees a 2nd-order approximation locally around $(\Delta x_1,\Delta x_2,\Delta y) = (0,0,0)$. That is, the higher-order terms in (M1) are $o(\|(\Delta x_1,\Delta x_2,\Delta y)\|^2)$ when the point $(\Delta x_1,\Delta x_2)$ moves to $(0,0)$ along any path in the coexistence region (see Fig. 1**e**, **f**).

One way to show the existence of the expansion (M1) is based on a milder regularity assumption. Indeed, in Ref. 1 it is postulated that the dimorphic fitness $s_{x_1,x_2}(y)$ has smooth directional derivatives at the singular point $(x_1,x_2) = (x^*,x^*)$ w.r.t. any direction $(w_1,w_2) := (\cos\theta,\sin\theta)$ in the coexistence region. The assumption is based on the ecological origin of the fitness function, that condones the regularity assumption to be applied to the attractor of coexistence. That is, the map from (x_1,x_2) to the attractor admits directional limits (and smooth derivatives) at (x^*,x^*), despite the map's discontinuity at (x^*,x^*)—population i being absent on the extinction boundary i and present along boundary j ($i = 1,2$, $j = 2,1$). This is so far shown to be the case (by direct computation of the directional limits) for the class of unstructured ecological models under stationary coexistence.[20]

Once the directional smoothness at $(x_1,x_2) = (x^*,x^*)$ is assumed, one should proceed as follows to show the kth-order differentiability (in the sense specified above) of the dimorphic fitness at $(x_1,x_2,y) = (x^*,x^*,x^*)$. Consider the restriction $\bar{s}(\varepsilon,\Delta y,w_1,w_2) := s_{x^*+\varepsilon w_1,x^*+\varepsilon w_2}(x^*+\Delta y)$ of the dimorphic fitness on the θ-ray ($x_i = x^*+\varepsilon w_i$, $i = 1,2$, $\varepsilon \geq 0$ being the distance of point (x_1,x_2) from (x^*,x^*)) and expand it jointly in $(\varepsilon,\Delta y)$ around $(\varepsilon,\Delta y) = (0,0)$:

$$\begin{aligned} \bar{s}(\varepsilon,\Delta y,w_1,w_2) &:= s_{x^*+\varepsilon w_1,x^*+\varepsilon w_2}(x^*+\Delta y) \\ &= s^* + \bar{s}_{10}(w_1,w_2)\varepsilon + \bar{s}_{01}\Delta y + \tfrac{1}{2}\bar{s}_{20}(w_1,w_2)\varepsilon^2 + \bar{s}_{11}(w_1,w_2)\varepsilon\Delta y + \tfrac{1}{2}\bar{s}_{02}\Delta y^2 + \cdots \end{aligned} \tag{M2}$$

Note the indexes of the expansion's coefficients, indicating the order of differentiation w.r.t. $(\varepsilon,\Delta y)$, and that the coefficients involving ε-derivatives are explicitly indicated as functions of the direction (w_1,w_2). Then, the dimorphic fitness is k^{th}-times differentiable at $(x_1,x_2,y) = (x^*,x^*,x^*)$ if the kth-order term in the expansion (M2) is polynomial of degree k in (w_1,w_2). More precisely, the kth-order coefficient $\bar{s}_{d\,k-d}(w_1,w_2)$ of the monomial $\varepsilon^d\Delta y^{k-d}$, $d \leq k$, must be either identically zero or a (w_1,w_2)-polynomial of degree d. Moreover, the higher-order terms in (M2) are $O(\|(\varepsilon,\Delta y)\|^3)$—because of the assumed directional smoothness—and they formally coincide with the higher-order terms in (M1)—except that (w_1,w_2) can change in (M1) along the path followed by $(\Delta x_1,\Delta x_2)$. The higher-order terms in (M1) are hence $O(\|(\Delta x_1,\Delta x_2,\Delta y)\|^3)$, implying the required little-o approximation.

Monomorphic fitness $s_x(y)$

zero order	$s^* := s_{x^*}(x^*) = 0$	(neutrality)	
1st order	$\partial_y s^* := \partial_y s_{x^*}(y)\vert_{y=x^*} = 0$	(singularity (1))	
2nd order	$\partial_{xy} s^* := \partial_{xy} s_x(y)\vert_{x=y=x^*} < 0$	(coexistence (2))	$\partial_{y^2} s^* := \partial_{y^2} s_{x^*}(y)\vert_{y=x^*}$
3rd order	$\partial_{x^2y} s^* := \partial_{x^2y} s_x(y)\vert_{x=y=x^*}$	$\partial_{xy^2} s^* := \partial_{xy^2} s_x(y)\vert_{x=y=x^*}$	$\partial_{y^3} s^* := \partial_{y^3} s_{x^*}(y)\vert_{y=x^*} \neq 0$ (genericity (7))
neutrality $s_x(x)=0$	$\partial_{x^k} s^* := \partial_{x^k} s_x(x^*)\vert_{x=x^*} = -\sum_{d=1}^{k} \binom{k}{d} \partial_{x^{k-d}y^d} s^*$		

Dimorphic fitness $s_{x_1,x_2}(y)$

zero order	$s^* = s_{x^*,x^*}(x^*) = 0$			
1st order	$s^*_{100} = s^*_{010} = 0$	$s^*_{001} = 0$		
2nd order	$s^*_{200} = s^*_{020} = 0$	$s^*_{110} = \frac{1}{2}\partial_{y^2} s^*$	$s^*_{101} = s^*_{011} = -\frac{1}{2}\partial_{y^2} s^*$	$s^*_{002} = \partial_{y^2} s^*$
3rd order	$s^*_{300} = s^*_{030} = 0$	$s^*_{210} = s^*_{120} = -\frac{1}{2}\frac{\partial_{y^2} s^* \partial_{xy^2} s^*}{\partial_{xy} s^*} + \frac{1}{3}\partial_{y^3} s^*$	$s^*_{201} = s^*_{021} = \frac{1}{2}\frac{\partial_{y^2} s^* \partial_{xy^2} s^*}{\partial_{xy} s^*} - \frac{1}{3}\partial_{y^3} s^*$	
	$s^*_{111} = \frac{1}{2}\frac{\partial_{y^2} s^* \partial_{xy^2} s^*}{\partial_{xy} s^*} - \frac{1}{6}\partial_{y^3} s^*$	$s^*_{102} = s^*_{012} = -\frac{1}{2}\frac{\partial_{y^2} s^* \partial_{xy^2} s^*}{\partial_{xy} s^*}$	$s^*_{003} = \partial_{y^3} s^*$	

Directional derivatives $\bar{s}_{d\,k-d}(w_1,w_2)$**,** $d>0$

1st order	$\bar{s}_{10}(w_1,w_2) = s^*_{100} w_1 + s^*_{010} w_2$	
2nd order	$\bar{s}_{20}(w_1,w_2) = s^*_{200} w_1^2 + 2 s^*_{110} w_1 w_2 + s^*_{020} w_2^2$	$\bar{s}_{11}(w_1,w_2) = s^*_{101} w_1 + s^*_{011} w_2$
3rd order	$\bar{s}_{30}(w_1,w_2) = s^*_{300} w_1^3 + 3 s^*_{210} w_1^2 w_2 + 3 s^*_{120} w_1 w_2^2 + s^*_{030} w_2^3$	$\bar{s}_{21}(w_1,w_2) = s^*_{201} w_1^2 + 2 s^*_{111} w_1 w_2 + s^*_{021} w_2^2$
	$\bar{s}_{12}(w_1,w_2) = s^*_{102} w_1 + s^*_{012} w_2$	

Extinction boundary 2 $s_{x_1}(x_2) = 0$

2nd order	$\tan\theta_2(0) = -\frac{2\partial_{xy} s^* + \partial_{y^2} s^*}{\partial_{y^2} s^*}$
3nd order	$\theta_2'(0) = -\frac{4(\partial_{xy} s^*)^2 \partial_{y^3} s^* - 2\partial_{xy} s^* \partial_{y^2} s^* (3\partial_{xy^2} s^* - \partial_{y^3} s^*) + (\partial_{y^2} s^*)^2 (3\partial_{x^2y} s^* + \partial_{y^3} s^*)}{6\sqrt{2}\left((\partial_{xy} s^*)^2 + (\partial_{xy} s^* + \partial_{y^2} s^*)^2\right)^{3/2}}$

Table. Notation and results summary

Unfortunately, without specific assumptions on the underlying ecological model—i.e., only exploiting the consistency relations C1–C3 of the dimorphic fitness—the above procedure works only up to $k=2$, as we now show (following unpublished lecture notes by J.A.J. Metz).

First note that the smoothness of the dimorphic fitness w.r.t. the mutant strategy y is granted at $(x_1,x_2,y) = (x^*,x^*,x^*)$ by the smoothness of the monomorphic fitness together with property C1. By C1 we can actually write

$$s^* = 0, \quad \bar{s}_{01} = \partial_y s^* = 0, \quad \bar{s}_{02} = \partial_{y^2} s^* \tag{M3}$$

(see Table, Monomorphic fitness, for the notation summary).

Imposing C3 (a and b), i.e., $\bar{s}(\varepsilon,\varepsilon w_1,w_1,w_2) = 0$ and $\bar{s}(\varepsilon,\varepsilon w_2,w_1,w_2) = 0$, and collecting from (M2) the resulting conditions at order ε and ε^2, give the following constraints

$$\bar{s}_{10}(w_1,w_2) + \bar{s}_{01} w_1 = 0, \quad \tfrac{1}{2}\bar{s}_{20}(w_1,w_2) + \bar{s}_{11}(w_1,w_2) w_1 + \tfrac{1}{2}\partial_{y^2} s^* w_1^2 = 0, \tag{M4a}$$

$$\bar{s}_{10}(w_1,w_2) + \bar{s}_{01} w_2 = 0, \quad \tfrac{1}{2}\bar{s}_{20}(w_1,w_2) + \bar{s}_{11}(w_1,w_2) w_2 + \tfrac{1}{2}\partial_{y^2} s^* w_2^2 = 0, \tag{M4b}$$

the first Eqs. in (M4a,b) yielding

$$\bar{s}_{10}(w_1,w_2) = 0 \tag{M5a}$$

by (M3), the second solving for

$$\bar{s}_{20}(w_1,w_2) \;=\; \partial_{y^2} s^* w_1 w_2, \tag{M5b}$$

$$\bar{s}_{11}(w_1,w_2) \;=\; -\tfrac{1}{2}\partial_{y^2} s^* (w_1 + w_2) \tag{M5c}$$

(under $w_1 \neq w_2$ in the coexistence region).

The identified functions $\bar{s}_{10}$, $\bar{s}_{20}$, $\bar{s}_{11}$ are indeed polynomial in (w_1, w_2) of the expected degree ($\bar{s}_{10}$ is identically zero, whereas $\bar{s}_{20}$ and $\bar{s}_{11}$ are of degree 2 and 1, respectively), proving the twice differentiability of the dimorphic fitness. Note that the functions are symmetric w.r.t. the diagonal $w_1 = w_2$, i.e., $\bar{s}_{d\,k-d}(w_1, w_2) = \bar{s}_{d\,k-d}(w_2, w_1)$, meaning that imposing C2 is redundant. The first- and second-order coefficients in the expansion (M1) are then determined by the standard linear combinations reported in Table (see Directional derivatives, 1st and 2nd orders, with results in Dimorphic fitness).

The consistency relations C1–C3 cannot determine the third and higher orders in the expansion (M2), because C3 gives only two constraints (C3a and C3b) among the k unknown functions $\bar{s}_{d\,k-d}(w_1, w_2)$, $d = 1, \ldots, k$ at order k. Again C2 is of no help in determining the unknown functions, it simply imposes the diagonal symmetry. To further constrain the coefficients of the expansion (M2) at order $k \geq 3$, a specific class of ecological models must be considered to allow the direct computation of the directional derivatives (as done in Ref. 20). Note that the monomorphic-dimorphic link is not fully exploited in C1, as it is valid also along the extinction boundaries (on which only one population is present). This is however of no help here, because the boundary in general is not straight (see Fig. 1**e**, **f**), so that, directionally, the link has only consequences at the singular point (x^*, x^*).

2 Expansion of the resident-mutant coexistence region

The extinction boundary i of the resident-mutant coexistence region, along which only the resident x_j is present ($i = 1, 2$, $j = 2, 1$) is defined by

$$s_{x_j}(x_i) = 0, \tag{M6}$$

the invasion fitness of strategy x_i being positive in the coexistence region and negative after crossing boundary i (Fig. 1**a**–**c**).

The two boundaries are evidently related by the symmetry w.r.t. the diagonal $x_1 = x_2$—one is obtained from the other by exchanging x_1 and x_2 in (M6)—so that below we focus on boundary 2. To approximate it locally to the singular point (x^*, x^*), we rewrite it in polar coordinates (ε, θ) as $\theta = \theta_2(\varepsilon)$, $\theta_2(\varepsilon)$ being the function that gives the angle θ of the boundary point at distance ε from (x^*, x^*). The function $\theta_2(\varepsilon)$ is implicitly defined by Eq. (9) of the main text (the boundary definition in polar coordinates, reported below)

$$s_{x_1}(x_2) = s_{x^* + \varepsilon \cos\theta_2(\varepsilon)}(x^* + \varepsilon \sin\theta_2(\varepsilon)) = 0, \tag{9}$$

which holds good for any (sufficiently small) $\varepsilon \geq 0$.

The approximation is in terms of an ε-expansion locally to $\varepsilon = 0$, i.e., $\theta_2(\varepsilon) = \theta_2(0) + \theta_2'(0)\varepsilon + \cdots + \theta_2^{(k)}(0)\varepsilon^k/k! + \cdots$, to be used also for negative ε to describe the boundary across the diagonal $x_1 = x_2$. The coefficients $\theta_2^{(k)}(0)$, $k \geq 0$, of the expansion can be obtained by solving the ε-derivatives of Eq. (9) at $\varepsilon = 0$. The first derivative turns out to be the identity due to the fitness neutrality $s_x(x) = 0$, whereas the second and third derivatives respectively solve for $\theta_2(0)$ and $\theta_2'(0)$. The result is reported in the Table (Extinction boundary 2), where only the monomorphic fitness derivatives with at least one order of derivation w.r.t. the mutant strategy are used—the pure x-derivatives $\partial_{x^k} s^*$ are avoided by exploiting the fitness neutrality (see Table, Monomorphic fitness). In general, the kth-order coefficient $\theta_2^{(k)}(0)$ is determined by the monomorphic fitness derivatives up to order $k+2$.

The angle $\theta_2(0)$ gives the tangent direction to the extinction boundary 2 at (x^*, x^*). Under the condition

$$\tan\theta_2(0) \neq 1, \quad \text{i.e.,} \quad \partial_{xy} s^* + \partial_{y^2} s^* \neq 0, \tag{M7}$$

which is met close to the ESS-branching transition ($\partial_{y^2} s^* \approx 0$ under the coexistence condition (2)), there are two solutions for $\theta_2(0)$, one in $(\frac{1}{4}\pi, \frac{5}{4}\pi)$ (above the diagonal) and the other at distance π in $(-\frac{3}{4}\pi, \frac{1}{4}\pi)$ (below the diagonal). They respectively give, for $\varepsilon \geq 0$, the boundary branch above and below the diagonal. We consider the former solution (the other option yielding same/opposite coefficients $\theta_2^{(k)}(0)$ for even/odd $k \geq 1$). Note that $\theta_2(0)$ decreases through $\frac{1}{2}\pi$ in the transition from ESS to branching (the opening angle $\theta_1(0) - \theta_2(0)$ of the coexistence region increases from acute to obtuse, see Fig. 1**e**, **f**). Also note that the coexistence condition (2) implies $\tan\theta_2(0) \neq -1$, i.e., $\theta_2(0) \neq \frac{3}{4}\pi$, so that the tangent direction to boundary 2 at (x^*, x^*) cannot be anti-diagonal.

The first derivative $\theta_2'(0)$ is nonzero close to the ESS-branching transition (under the coexistence and genericity conditions (2) and (7)) and determines the local curvature of the boundary—whether θ increases or decreases while moving away from (x^*, x^*).

The extinction boundaries 1 and 2 in Fig. 1**e**, **f** are produced with the truncations

$$\theta_1(\varepsilon) = \tfrac{3}{2}\pi - \theta_2(0) + \theta_2'(0)\varepsilon, \quad \theta_2(\varepsilon) = \theta_2(0) + \theta_2'(0)\varepsilon, \tag{M8}$$

involving up to 3rd-order monomorphic fitness derivatives. Note that the diagonal symmetry yields for boundary 1 $\theta_1(0) = \frac{3}{2}\pi - \theta_2(0)$ and $\theta_1^{(k)}(0) = (-1)^{k-1}\theta_2^{(k)}(0)$, $k \geq 1$.

3 Expansion of the dimorphic invasion fitness

We now assume that the dimorphic fitness $s_{x_1,x_2}(y)$ is three-times differentiable at $(x_1,x_2,y)=(x^*,x^*,x^*)$, in the sense specified in Sect.1. As stated in the main text, we recall once more that this is so far shown to be the case only for the class of unstructured ecological models under stationary coexistence (done in Ref. 20 by direct computation of the directional expansion (M2)), though we expect the assumption to hold good for any class of ecological models yielding a smooth monomorphic fitness for a one-dimensional strategy.

We hence assume the existence of the 3rd-order local expansion

$$\begin{aligned} s_{x_1,x_2}(y) &= s^* + s^*_{100}\Delta x_1 + s^*_{010}\Delta x_2 + s^*_{001}\Delta y \\ &+ \tfrac{1}{2}s^*_{200}\Delta x_1^2 + s^*_{110}\Delta x_1\Delta x_2 + s^*_{101}\Delta x_1\Delta y + \tfrac{1}{2}s^*_{020}\Delta x_2^2 + s^*_{011}\Delta x_2\Delta y + \tfrac{1}{2}s^*_{002}\Delta y^2 \\ &+ \tfrac{1}{6}s^*_{300}\Delta x_1^3 + \tfrac{1}{2}s^*_{210}\Delta x_1^2\Delta x_2 + \tfrac{1}{2}s^*_{201}\Delta x_1^2\Delta y + \tfrac{1}{2}s^*_{120}\Delta x_1\Delta x_2^2 + s^*_{111}\Delta x_1\Delta x_2\Delta y + \tfrac{1}{2}s^*_{102}\Delta x_1\Delta y^2 \\ &+ \tfrac{1}{6}s^*_{030}\Delta x_2^3 + \tfrac{1}{2}s^*_{021}\Delta x_2^2\Delta y + \tfrac{1}{2}s^*_{012}\Delta x_2\Delta y^2 + \tfrac{1}{6}s^*_{003}\Delta y^3 + o(\|(\Delta x_1,\Delta x_2,\Delta y)\|^3), \end{aligned} \tag{M9}$$

for $(\Delta x_1,\Delta x_2)$ within the coexistence region, and we determine the expansion's coefficients by applying the consistency relations C1–C3.

Applying C2 implies the symmetry constraints $s^*_{d_1\,d_2\,k-d_1-d_2} = s^*_{d_2\,d_1\,k-d_1-d_2}$, $d_1+d_2\le k$, at order k. We therefore eliminate the unknown coefficients with $d_1<d_2$ and write the constraints implied by C1 and C3 in the 12 unknowns with $d_1\ge d_2$ at orders $k=1,2,3$, plus the zero-order coefficient s^* (see Table, Dimorphic fitness). So doing, we also eliminate the relation C3b, as it is implied by C2 and C3a.

Applying C1 we get, analogously to (M3),

$$s^*=0,\quad s^*_{001}=\partial_y s^*=0,\quad s^*_{002}=\partial_{y^2}s^*,\quad s^*_{003}=\partial_{y^3}s^*, \tag{M10}$$

so we remiain with 9 unknowns, s^*_{100} at 1st order, s^*_{200}, s^*_{110}, s^*_{101} at 2nd order, and s^*_{300}, s^*_{210}, s^*_{201}, s^*_{111}, s^*_{102} at 3rd order.

Applying C3a, i.e., substituting $\Delta y=\Delta x_1$ in the truncated expansion (M9) and equating to zero the coefficient of each monomial, we get the following linear constraints, where the results in (M10) are already taken into account:

$$\Delta x_1:\quad s^*_{100}=0, \tag{M11a}$$

$$\Delta x_2:\quad s^*_{100}=0, \tag{M11b}$$

$$\Delta x_1^2:\quad \tfrac{1}{2}s^*_{200}+s^*_{101}+\tfrac{1}{2}\partial_{y^2}s^*=0, \tag{M11c}$$

$$\Delta x_1\Delta x_2:\quad s^*_{110}+s^*_{101}=0, \tag{M11d}$$

$$\Delta x_2^2:\quad \tfrac{1}{2}s^*_{200}=0, \tag{M11e}$$

$$\Delta x_1^3:\quad \tfrac{1}{6}s^*_{300}+\tfrac{1}{2}s^*_{201}+\tfrac{1}{2}s^*_{102}+\tfrac{1}{6}\partial_{y^3}s^*=0, \tag{M11f}$$

$$\Delta x_1^2\Delta x_2:\quad \tfrac{1}{2}s^*_{210}+s^*_{111}+\tfrac{1}{2}s^*_{102}=0, \tag{M11g}$$

$$\Delta x_1\Delta x_2^2:\quad \tfrac{1}{2}s^*_{210}+\tfrac{1}{2}s^*_{201}=0, \tag{M11h}$$

$$\Delta x_2^3:\quad \tfrac{1}{6}s^*_{300}=0. \tag{M11i}$$

The constraints at orders 1 and 2 are 5, but only 4 are independent (the first two are identical) and solve for the 4 unknowns, giving the same results obtained in Sect.1 (see Table, Dimorphic fitness). The constraints at order 3 are however 4 for 5 unknown coefficients.

To find the missing constraint, we now exploit the monomorphic-dimorphic link along the extinction boundary 2, on which only population 1 is present. This also implies, by the boundaries' symmetry and property C2, the link on the extinction boundary 1. Using the polar characterization of the boundary introduced in Sect. 2, we thus replace property C1 with

$$\text{C1}':\; s_{x^*+\varepsilon\cos\theta_2(\varepsilon),\,x^*+\varepsilon\sin\theta_2(\varepsilon)}(x^*+\Delta y) = s_{x^*+\varepsilon\cos\theta_2(\varepsilon)}(x^*+\Delta y),$$

which holds good for any (sufficiently small) $\varepsilon\ge 0$ and Δy.

Here is where we really need the differentiability of the dimorphic fitness. To exploit C1$'$ and constrain the coefficients of the expansion (M9), we need to impose the $(\varepsilon,\Delta y)$-derivatives of C1$'$ at $(\varepsilon,\Delta y)=(0,0)$. Such derivatives formally involve the partial derivatives of the dimorphic fitness at $(x_1,x_2,y)=(x^*,x^*,x^*)$, that are not defined. Equivalently, we can substitute the truncated expansion (M9) in the left-hand side of C1$'$ and then differentiate. So doing, we obtain the following linear

constraints, organized by the order of the considered derivative:

$$1: \quad s^* = 0 \tag{M12a}$$

$$\varepsilon: \quad s^*_{100} = 0, \tag{M12b}$$

$$\Delta y: \quad s^*_{001} = \partial_y s^* = 0, \tag{M12c}$$

$$\varepsilon^2: \quad 2\big(2(\partial_{xy}s^*)^2 + 2\partial_{xy}s^*\partial_{y^2}s^* + (\partial_{y^2}s^*)^2\big)s^*_{200} - 2(2\partial_{xy}s^* + \partial_{y^2}s^*)\partial_{y^2}s^*s^*_{110} + (2\partial_{xy}s^* + \partial_{y^2}s^*)(\partial_{y^2}s^*)^2 = 0, \tag{M12d}$$

$$\varepsilon\Delta y: \quad s^*_{101} = -\tfrac{1}{2}\partial_{y^2}s^*, \tag{M12e}$$

$$\Delta y^2: \quad s^*_{002} = \partial_{y^2}s^*, \tag{M12f}$$

$$\begin{aligned}\varepsilon^3: \quad & 4\partial_{xy}s^*(\partial_{xy}s^* + \partial_{y^2}s^*)\big(4(\partial_{xy}s^*)^2\partial_{y^3}s^* - 2\partial_{xy}s^*\partial_{y^2}s^*(3\partial_{xy^2}s^* - \partial_{y^3}s^*) + (\partial_{y^2}s^*)^2(3\partial_{x^2y}s^* + \partial_{y^3}s^*)\big)s^*_{200} \\ & -2\partial_{xy}s^*\partial_{y^2}s^*\big(8(\partial_{xy}s^*)^3 + 16(\partial_{xy}s^*)^2\partial_{y^2}s^* + 12\partial_{xy}s^*(\partial_{y^2}s^*)^2 + 3(\partial_{y^2}s^*)^3\big)s^*_{300} \\ & +6\partial_{xy}s^*(2\partial_{xy}s^* + \partial_{y^2}s^*)^2(\partial_{y^2}s^*)^2s^*_{210} - (2\partial_{xy}s^* + \partial_{y^2}s^*)^2(\partial_{y^2}s^*)^2(2\partial_{xy}s^*\partial_{y^3}s^* - 3\partial_{y^2}s^*\partial_{xy^2}s^*) = 0,\end{aligned} \tag{M12g}$$

$$\begin{aligned}\varepsilon^2\Delta y: \quad & 6\big(2(\partial_{xy}s^*)^2 + 2\partial_{xy}s^*\partial_{y^2}s^* + (\partial_{y^2}s^*)^2\big)s^*_{201} - 6(2\partial_{xy}s^* + \partial_{y^2}s^*)\partial_{y^2}s^*s^*_{111} \\ & +\big(4(\partial_{xy}s^*)^2\partial_{y^3}s^* - 2\partial_{xy}s^*\partial_{y^2}s^*(3\partial_{xy^2}s^* - \partial_{y^3}s^*) + (\partial_{y^2}s^*)^2\partial_{y^3}s^*\big) = 0,\end{aligned} \tag{M12h}$$

$$\varepsilon\Delta y^2: \quad 2\partial_{xy}s^*s^*_{102} + \partial_{y^2}s^*\partial_{xy^2}s^* = 0, \tag{M12i}$$

$$\Delta y^3: \quad s^*_{003} = \partial_{y^3}s^*. \tag{M12j}$$

Of course the constraints in (M12) include those in (M10), obtained by C1 at order 0 and with the pure Δy-derivatives. For the ε- and mixed-derivatives, the results at 1st order (M12b,c) are exploited to obtain the constraints at 2nd order and the results at 1st and 2nd orders (M12b–f) are exploited to obtain the constraints at 3rd order. This allows to eliminate $\theta_2'(0)$ at 2nd order and $\theta_2''(0)$ at 3rd order, so that only the expressions for $\theta_2(0)$ and $\theta_2'(0)$ (Table, Extinction boundary 2) are involved and substituted where needed. Also the coexistence condition (2) is taken into account to remove the denominators coming from $\theta_2(0)$ and $\theta_2'(0)$.

The constraints implied by C1′ up to 2nd order (M12a–f) are obviously redundant w.r.t. those implied by C1–C3 in (M10) and (M11). By contrast, each of the new 3rd-order constraints (M12g–i) equivalently solves, together with (M11f–i), for the five 3rd-order coefficients in (M9). The results are reported in the Table (Dimorphic fitness, 3rd order). Note that, under our smoothness assumption for the dimorphic fitness, the 3rd-order coefficients determine (according to the linear combinations in Table, Directional derivatives) the directional functions $\bar{s}_{30}(w_1,w_2)$, $\bar{s}_{21}(w_1,w_2)$, $\bar{s}_{12}(w_1,w_2)$ appearing at order 3 in the directional expansion (M2). The results indeed coincide with those found for the class of unstructured ecological models under stationary coexistence.[20]

Unfortunately, the constraints implied by properties C1′-C3 at 4th order are not enough to solve for the 15 4th-order coefficients of the expansion (M9). Out of the 16 constraints, only 14 are independent. In general, we have $(k+1)(k+2)/2$ coefficients at order k and the number of constraints, counting redundancies, is $k+1$ for C1′ and C3a and $(k/2)(k/2+1)$ (k even) or $(k+1)^2/4$ (k odd) for C2. Thus, even counting redundancies, the number of unknowns exceeds the number of constraints starting from order 6. This does not necessarily mean that the geometry of the dimorphic fitness, locally to the singularity $(x_1,x_2,y) = (x^*,x^*,x^*)$, is not fully determined by the local geometry of the monomorphic fitness. The two fitness functions are linked to each other by the underlying ecological model, that is a much stronger link than C1′. Only by exploiting the full ecological link we can then answer the above question, starting from order 4. Whether the answer is yes or no remains an open theoretical issue of AD.

4 The canonical model of the ESS-branching transition

Using the results derived in Sects. 2 and 3, we now derive the canonical model (8). We consider the continuous evolutionary dynamics ruled by the so-called AD *canonical equation*[11,12] in the limit of rare and infinitesimally small mutational steps. We note however that the assumption of rare mutation can be relaxed[14] and that the dynamics of model (8) (the direction of evolution in Eq. (8a), the ecological scaling rates in Eqs. (8b,c), and the fitness gradients in Eqs. (8d,e)) inform as well about the adaptive dynamics driven by sufficiently small but finite strategy mutations.[23,24]

In the simple setting of unstructured ecological models under stationary coexistence, the AD canonical equation reads

$$\dot{x} = \tfrac{1}{2}\mu(x)\sigma(x)^2\bar{n}(x)\partial_y s_x(y)|_{y=x} \tag{M13}$$

before branching (monomorphic phase) and

$$\dot{x}_i = \tfrac{1}{2}\mu(x_i)\sigma(x_i)^2\bar{n}_i(x_1,x_2)\partial_y s_{x_1,x_2}(y)|_{y=x_i}, \quad i = 1,2, \tag{M14}$$

after branching (dimorphic phase), where $\mu(x)$ and $\sigma(x)^2$ respectively scale with the frequency and variance of mutations in strategy x (half of which are at disadvantage and go extinct), $\bar{n}(x)$ and $\bar{n}_i(x_1,x_2)$ are the resident equilibrium densities (see

Fig. 1**c**), and $\dot{x}$ is the time-derivative of x on an evolutionary time scale that is fully separated from the ecological one. In more complex ecological settings—including structured population models and non-stationary attractors of coexistence—the fitness gradients in Eqs. (M13) and (M14) are scaled by the average birth outputs shown by the populations at the ecological regime,[11,12,18] in lieu of the resident equilibrium densities. This ecological scaling has been shown equivalent to four times the *effective population size*,[35] as defined in population genetics.

As an approximation of the stochastic dynamics driven by finite mutations, Eqs. (M13) and (M14) give better results the more mutations are small. The convergence of the stochastic dynamics to the deterministic solution, as mutational steps become infinitesimal, is however non-uniform,[12] i.e., closer to a singular strategy the approximation requires smaller mutations. As a consequence, branching must be discussed by resorting on finite mutations close to the singular strategy[36] (see also the discussion on finite mutations following Fig. 2 in the main text).

To derive the canonical model (8), we first get rid of the evolutionary scaling $\frac{1}{2}\mu(x_i)\sigma(x_i)^2$ in Eq. (M14). Locally to the singular point (x^*,x^*) this can be done in two steps. A near-identity coordinate transformation, $z_i = z_i(x_1,x_2)$, $i=1,2$ (near-identity meaning $\partial z_i/\partial x_j = 1$ if $i=j$, 0 otherwise), whose expansion can be set to eliminate all the derivatives of μ and σ in the expansion of the scaling factor around $x_1 = x_2 = x^*$; a time-scaling $\tau = \frac{1}{2}\mu(x^*)\sigma(x^*)^2 t$, τ being the new time. For simplicity, we keep on using variables x_i (actually Δx_i) and t for the new variables and time.

Second, we approximate the ecological scaling factor in Eq. (M14). To avoid specific assumptions at the ecological level, the idea is to replace the resident equilibrium density $\bar{n}_i(x_1,x_2)$ with a quantity that is determined by the geometry of the monomorphic fitness and that qualitatively behaves as the birth output of population i, $i=1,2$, locally to the singular point (x^*,x^*). So doing, we lose the quantitative mapping between our canonical model and the AD canonical equation (M14), that can however be saved only working with a specific class of ecological models.

The birth output of population i is positive in the resident-mutant coexistence region and vanishes while approaching the extinction boundary i. It is therefore sign-equivalent, within the coexistence region (boundaries included), to the invasion fitness of strategy x_i that defines boundary i in (M6). However, while the monomorphic fitness is smooth and quadratically vanishes at the singular point (x^*,x^*), the birth output i is discontinuous at (x^*,x^*)—population i being absent on boundary i and present along boundary j, $i \neq j$.

Expanding the fitnesses of strategies x_1 (against x_2) and x_2 (against x_1) around (x^*,x^*) we get

$$s_{x_2}(x_1) = s_{x^*+\Delta x_2}(x^*+\Delta x_1) = \eta_1(\Delta x_1,\Delta x_2)(\Delta x_1-\Delta x_2) + O(\|(\Delta x_1,\Delta x_2)\|^4), \tag{M15a}$$

$$s_{x_1}(x_2) = s_{x^*+\Delta x_1}(x^*+\Delta x_2) = \eta_2(\Delta x_1,\Delta x_2)(\Delta x_2-\Delta x_1) + O(\|(\Delta x_1,\Delta x_2)\|^4), \tag{M15b}$$

with the quantities $\eta_1(\Delta x_1,\Delta x_2)$ and $\eta_2(\Delta x_1,\Delta x_2)$ defined in Eqs. (8b,c) of the main text (reported below)

$$\eta_1(\Delta x_1,\Delta x_2) := \partial_{xy}s^*\Delta x_2 + \tfrac{1}{2}\partial_{y^2}s^*(\Delta x_1+\Delta x_2) + \tfrac{1}{2}\partial_{x^2y}s^*\Delta x_2^2 + \tfrac{1}{2}\partial_{xy^2}s^*\Delta x_2(\Delta x_1+\Delta x_2) + \tfrac{1}{6}\partial_{y^3}s^*(\Delta x_1^2+\Delta x_1\Delta x_2+\Delta x_2^2), \tag{8b}$$

$$\eta_2(\Delta x_1,\Delta x_2) := \eta_1(\Delta x_2,\Delta x_1). \tag{8c}$$

We note that the following expressions

$$\tilde{n}_1(\Delta x_1,\Delta x_2) := -\frac{\bar{n}^*}{\partial_{xy}s^*}\frac{\eta_1(\Delta x_1,\Delta x_2)}{\Delta x_1-\Delta x_2}, \tag{M16a}$$

$$\tilde{n}_2(\Delta x_1,\Delta x_2) := -\frac{\bar{n}^*}{\partial_{xy}s^*}\frac{\eta_2(\Delta x_1,\Delta x_2)}{\Delta x_2-\Delta x_1} = \tilde{n}_1(\Delta x_2,\Delta x_1), \tag{M16b}$$

are also sign-equivalent to the birth outputs of populations 1 and 2, respectively, and similarly behave discontinuously at $(\Delta x_1,\Delta x_2) = (0,0)$. Specifically, using the quadratic approximations $\eta_1(\Delta x_1,\Delta x_2) = 0$ and $\eta_2(\Delta x_2,\Delta x_1) = 0$ of the extinction boundaries 1 and 2—involving up to 3rd-order monomorphic fitness derivatives—the quantity $\tilde{n}_i(\Delta x_1,\Delta x_2)$ is zero along boundary i and its limit to (x^*,x^*) along boundary j is equal to the ecological scaling factor $\bar{n}^*$. To compute the latter limit we have ε-expanded (in polar coordinates) $\tilde{n}_i(\varepsilon\cos\theta,\varepsilon\sin\theta)$ at $\varepsilon=0$ along the boundary $\theta = \theta_j(\varepsilon)$ defined by $\eta_j(\varepsilon\cos\theta_j(\varepsilon),\varepsilon\sin\theta_j(\varepsilon)) = 0$. Note that this is a different boundary approximation than the truncations in (M8), that however gives the same angle $\theta_2(0)$ and curvature $\theta_2'(0)$ reported in Table (Extinction boundary 2). The result is indeed

$$\tilde{n}_i(\varepsilon\cos\theta_j(\varepsilon),\varepsilon\sin\theta_j(\varepsilon)) = \bar{n}^* + O(\varepsilon), \quad i=1,2,\ j=2,1. \tag{M17}$$

We thus replace in Eq. (M14) the resident equilibrium density $\bar{n}_i(x_1,x_2)$ with $\tilde{n}_i(\Delta x_1,\Delta x_2)$ from (M16a,b), arbitrarily setting the scaling factor $\bar{n}^*$ to 1. In the simple setting (unstructured ecological models under stationary coexistence), $\tilde{n}_i(\Delta x_1,\Delta x_2)$ was shown to correspond to the directional expansion (ε-expansion with $(\Delta x_1,\Delta x_2) = (\varepsilon\cos\theta,\varepsilon\sin\theta)$) of the equilibrium density $\bar{n}_i(x_1,x_2)$ up to the linear terms in $\eta_i(\Delta x_1,\Delta x_2)$, $i=1,2$.[20]

Third step, we compute the fitness gradient $\partial_y s_{x_1,x_2}(y)|_{y=x_i}$ using our expansion (4, 6), thus obtaining

$$\partial_y s_{x_1,x_2}(y)|_{y=x_i} = s_i(\Delta x_1,\Delta x_2)(\Delta x_i-\Delta x_j), \quad i=1,2,\ j=2,1, \tag{M18}$$

with the quantities $s_1(\Delta x_1,\Delta x_2)$ and $s_2(\Delta x_1,\Delta x_2)$ defined in Eqs. (8d,e) of the main text (reported below)

$$s_1(\Delta x_1,\Delta x_2) := \tfrac{1}{2}\partial_{y^2}s^* - \tfrac{1}{4}\frac{\partial_{y^2}s^*\partial_{xy^2}s^*}{\partial_{xy}s^*}(\Delta x_1+\Delta x_2)+\tfrac{1}{6}\partial_{y^3}s^*(2\Delta x_1+\Delta x_2), \tag{8d}$$

$$s_2(\Delta x_1,\Delta x_2) := s_1(\Delta x_2,\Delta x_1). \tag{8e}$$

Our canonical model of the ESS-branching transition then reads

$$\dot{x}_i = \tilde{n}_i(\Delta x_1,\Delta x_2)\,\partial_y s_{x_1,x_2}(y)|_{y=x_i} = -\frac{1}{\partial_{xy}s^*}\eta_i(\Delta x_1,\Delta x_2)\,s_i(\Delta x_1,\Delta x_2), \quad i=1,2, \tag{8a}$$

and is defined in the coexistence region delimited by the extinction boundaries $\eta_1(\Delta x_1,\Delta x_2)=0$ (boundary 1) and $\eta_2(\Delta x_1,\Delta x_2)=0$ (boundary 2). Note the cancellation of the differences $(\Delta x_i-\Delta x_j)$ at denominator in Eqs. (M16a,b) and at numerator in Eq. (M18), that makes the model equations polynomial (and therefore smooth!).

Close to ESS-branching transition ($\partial_{y^2}s^*\approx 0$ under the coexistence and genericity conditions (2) and (7)), model (8) is qualitatively equivalent to the dimorphic canonical equation (M14), locally to the singular point (x^*,x^*). Note that model (8) is not a $(\Delta x_1,\Delta x_2)$-expansion of the dimorphic canonical equation (M14). Even in the simple setting (unstructured ecological models under stationary coexistence) in which $\tilde{n}_i(\Delta x_1,\Delta x_2)$ quantitatively approximates the resident equilibrium density $\bar{n}_i(x_1,x_2)$, the canonical model misses some cubic terms—e.g. those given by the product of a linear term in $\eta_i(\Delta x_1,\Delta x_2)$ with the missing quadratic terms in $s_i(\Delta x_1,\Delta x_2)$. This is due to the choice of separately approximating the resident equilibrium densities and the fitness gradients, with the advantage of preserving some structural features of the canonical equation. E.g., the presence of boundary evolutionary equilibria when $\eta_i(\Delta x_1,\Delta x_2)$ and $s_j(\Delta x_1,\Delta x_2)$ vanish with $i\neq j$ and the ($O((\partial_{y^2}s^*)^2)$-approximated) connections of the flow nullclines with the horizontal and vertical extremal points of the extinction boundaries (see Fig. 2; see Ref. 1, Sect. 6.3.3, and Ref. 25, Appx., for further detail on the above structural features).

The unfolding parameter of the ESS-branching transition—that we move across zero—is $\partial_{y^2}s^*$. Four other parameters are left in model (8): $\partial_{xy}s^*$, $\partial_{x^2y}s^*$, $\partial_{xy^2}s^*$, $\partial_{y^3}s^*$, i.e., all second and third independent derivatives of the monomorphic fitness—the pure x-derivatives $\partial_{x^2}s^*$ and $\partial_{x^3}s^*$ being related to the others by the fitness neutrality (see Table, Monomorphic fitness). The first and last parameters are constrained by the coexistence and genericity conditions (2) and (7), whereas $\partial_{x^2y}s^*$ and $\partial_{xy^2}s^*$ play no role in the transition. In fact, though they both appear in $\eta_i(\Delta x_1,\Delta x_2)$ (see Eqs. (8b,c)) and only $\partial_{xy^2}s^*$ appears in $s_i(\Delta x_1,\Delta x_2)$ (see Eqs. (8d,e)), at the transition ($\partial_{y^2}s^*=0$) the curvature of the extinction boundaries and the flow nullclines are unaffected (see the expression of $\theta_2'(0)$ in Table, Extinction boundary 2, where the effect of $\partial_{x^2y}s^*$ and $\partial_{xy^2}s^*$ is modulated by $\partial_{y^2}s^*$, and note that $\partial_{xy^2}s^*$ is multiplied by $\partial_{y^2}s^*$ in $s_i(\Delta x_1,\Delta x_2)$), whereas the flow of model (8) is perturbed only in the cubic $(\Delta x_1,\Delta x_2)$-terms. With $\partial_{xy}s^*$ constrained in sign, $\partial_{y^3}s^*$ is the only relevant coefficient of the canonical model.

Finally, we note that model (8) serves as canonical model for the ESS-branching transition also in the presence of other evolving (one-dimensional) strategies (e.g. the transition to a higher polymorphism or the coevolution in a multi-species model). Whether the other strategies are at singularity or slowly evolving,[37] our analysis in Ref. 20, Appx. C, shows that they remain selectively neutral during the initial phase of branching in strategy x.

5 Unfolding of the ESS-branching transition

We analyze in this last section the dynamics of the canonical model (8), under the coexistence and genericity conditions (2) and (7), while varying the unfolding parameter $\partial_{y^2}s^*$ across zero. Model (8) is defined only within the resident-mutant coexistence region defined by $\tilde{n}_i(\Delta x_1,\Delta x_2)\geq 0$, $i=1,2$, see Eqs. (M16a,b) and (8b,c). However, for the purpose of the mathematical analysis, we extend the validity of the model's equations in a full neighborhood of $(\Delta x_1,\Delta x_2)=(0,0)$.

By inspection of Eq. (8a), it is easy to see that there are four equilibria (the pairs $(\Delta x_1,\Delta x_2)$ in a full neighborhood of $(0,0)$ at which $\dot{x}_1=\dot{x}_2=0$):

E0: $(\Delta x_1,\Delta x_2)=(0,0)$ zeroing η_1 and η_2.

E1: $(\Delta x_1,\Delta x_2)=(\Delta x_1^*,\Delta x_2^*)$ zeroing η_1 and s_2, with

$$\Delta x_1^* := -\frac{3}{\partial_{y^3}s^*}\partial_{y^2}s^* - \frac{9}{2}\frac{\partial_{xy^2}s^*}{\partial_{xy}s^*(\partial_{y^3}s^*)^2}(\partial_{y^2}s^*)^2 - \frac{9}{4}\frac{(3\partial_{xy^2}s^*-2\partial_{y^3}s^*)\partial_{xy^2}s^*}{(\partial_{xy}s^*)^2(\partial_{y^3}s^*)^3}(\partial_{y^2}s^*)^3 + O((\partial_{y^2}s^*)^4),$$

$$\Delta x_2^* := -\frac{9}{4}\frac{\partial_{xy^2}s^*}{(\partial_{xy}s^*\partial_{y^3}s^*)^2}(\partial_{y^2}s^*)^3 + O((\partial_{y^2}s^*)^4),$$

here expressed as power series in the unfolding parameter $\partial_{y^2}s^*$.

E2: $(\Delta x_1,\Delta x_2)=(\Delta x_2^*,\Delta x_1^*)$ symmetrically zeroing η_2 and s_1.

E3: $(\Delta x_1,\Delta x_2)=(\Delta x^*,\Delta x^*)$, $\Delta x^* := -\frac{\partial_{xy}s^*\partial_{y^2}s^*}{\partial_{xy}s^*\partial_{y^3}s^* - \partial_{y^2}s^*\partial_{xy^2}s^*}$, zeroing s_1 and s_2.

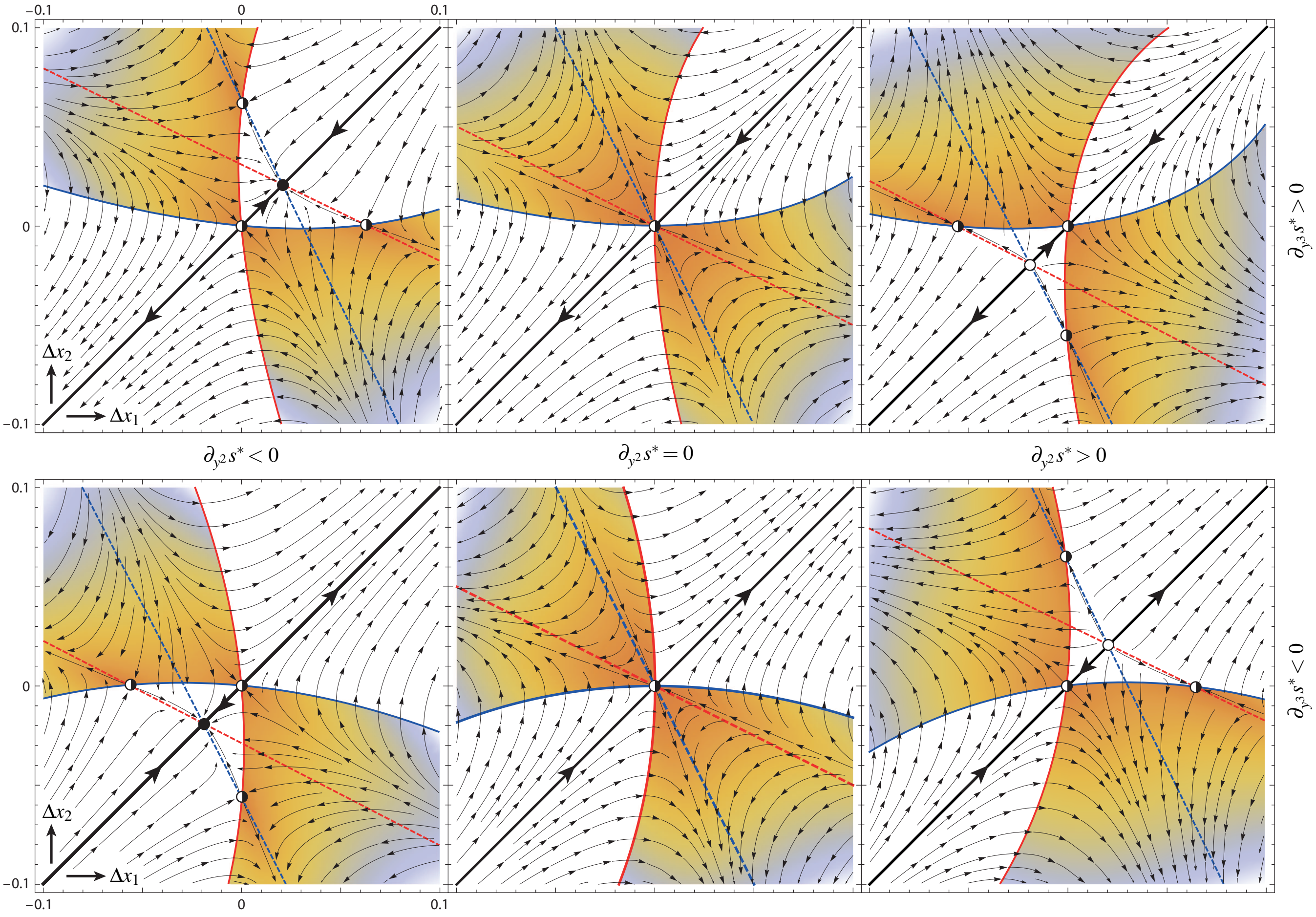

Figure M1. Unfolding of the ESS-branching transition according to model (8). The unfolding parameter $\partial_{y^2} s^*$ increases from left to right and vanishes in the central panel, in which all equilibria E0–E3 collide at $(\Delta x_1, \Delta x_2) = (0,0)$. Top row: $\partial_{y^3} s^* = 10$; bottom row: $\partial_{y^3} s^* = -10$; other parameters as in Fig. 2. The resident-mutant coexistence region is shaded, with color code orange-to-blue measuring the magnitude of the vector field. The extinction boundary 1 ($\eta_1(\Delta x_1, \Delta x_2) = 0$) and the x_1-nullcline ($s_1(\Delta x_1, \Delta x_2) = 0$) are plotted in blue (solid and dashed); red for boundary 2 ($\eta_2(\Delta x_1, \Delta x_2) = 0$) and the x_2-nullcline ($s_2(\Delta x_1, \Delta x_2) = 0$). At the transition (central panel) the nullclines pass through the singular point $(\Delta x_1, \Delta x_2) = (0,0)$ with slopes -2 and $-1/2$, respectively (see Eqs. (8d,e) with $\partial_{y^2} s^* = 0$ and $\partial_{y^3} s^* \neq 0$). Full points: attractors; half-filled points: saddles; empty points: repellors.

Note that E1 and E2 are symmetric boundary equilibria, respectively lying on the extinction boundaries 1 and 2, while E3 lies on the diagonal (and is therefore unfeasible for the evolutionary dynamics).

The four equilibria are all involved in the ESS-branching transition occurring at $\partial_{y^2} s^* = 0$, as they collide at $(0,0)$ at the transition. Under conditions (2) and (7), equilibria E0–E3 intersect transversely as $\partial_{y^2} s^*$ moves across zero. The transition classifies as a *non-simple branch point bifurcation*[38–40] (not to be confused with the branching point of AD!), i.e., the transversal intersection of more than two $\partial_{y^2} s^*$-parameterized equilibrium branches. This bifurcation generically requires the *continuation problem*[41] that defines the intersecting equilibrium branches to have a nullspace with dimension larger than two at the bifurcation. Specifically, the continuation problem is defined in the space $(\Delta x_1, \Delta x_2, \partial_{y^2} s^*)$ by

$$C(\Delta x_1, \Delta x_2, \partial_{y^2} s^*) := \begin{bmatrix} \eta_1(\Delta x_1, \Delta x_2, \partial_{y^2} s^*)\, s_1(\Delta x_1, \Delta x_2, \partial_{y^2} s^*) \\ \eta_2(\Delta x_1, \Delta x_2, \partial_{y^2} s^*)\, s_2(\Delta x_1, \Delta x_2, \partial_{y^2} s^*) \end{bmatrix} = 0,$$

where $\partial_{y^2} s^*$ is explicitly mentioned as an argument of η_i and s_i. The Jacobian of the (vector-valued) function C w.r.t. $(\Delta x_1, \Delta x_2, \partial_{y^2} s^*)$ is indeed a (2×3) null matrix at the bifurcation (easy to check), i.e., the nullspace is three-dimensional. Due to the diagonal symmetries of the canonical model (8), this bifurcation occurs with *codimension-one*, i.e., moving a single unfolding parameter (see Ref. 38, Sect. 8.2, for further detail).

Two cases can be distinguished, namely $\partial_{y^3} s^* > 0$ and $\partial_{y^3} s^* < 0$, whose unfoldings are pictured in Fig. M1 (top and bottom panels, respectively). The movements and stability of the four equilibria, as $\partial_{y^2} s^*$ goes from negative to positive, are evident

from the graphics (left-to-right panels; the trajectories of the canonical model (8) are drawn also outside the resident-mutant coexistence region to make stability easily readable).

Note that the stability for the unrestricted model is different from the stability for the dimorphic canonical equation. E.g., equilibrium E0 is always unstable (saddle type) for the unrestricted model. The associated Jacobian,

$$\tfrac{1}{2}\partial_{y^2}s^* \begin{bmatrix} \tfrac{1}{2}\partial_{y^2}s^* & \partial_{xy}s^*+\tfrac{1}{2}\partial_{y^2}s^* \\ \partial_{xy}s^*+\tfrac{1}{2}\partial_{y^2}s^* & \tfrac{1}{2}\partial_{y^2}s^* \end{bmatrix},$$

has eigenvalues $\frac{1}{2}\partial_{y^2}s^*\left(\partial_{xy}s^*+\partial_{y^2}s^*\right)$ and $-\frac{1}{2}\partial_{xy}s^*\partial_{y^2}s^*$ with the diagonal $\Delta x_1 = \Delta x_2$ and the anti-diagonal $\Delta x_1 + \Delta x_2 = 0$ as eigenvectors (away from the bifurcation, $\partial_{y^2}s^* \neq 0$). By contrast, E0 is stable/unstable for the dimorphic canonical equation when $\partial_{y^2}s^* \lessgtr 0$ (ESS/branching).

Also note that the two cases ($\partial_{y^3}s^* \gtrless 0$) are topologically equivalent, since at the transition there is, locally to (x^*, x^*), a symmetry w.r.t. the anti-diagonal. The distinction between the two cases is hence mathematically irrelevant. However, the distinction is biologically important, due to the different curvatures of the extinction boundaries in the two cases (the value of $\theta_2'(0)$, see Table, Extinction boundary 2, gives the curvature of the locally vertical boundary, negative/positive for $\partial_{y^3}s^* \gtrless 0$, see Fig. M1). Indeed, $\partial_{y^3}s^* \gtrless 0$ makes branching possible at the transition only for mutants with larger/smaller trait values (as already noted, without a formal derivation, in Ref. 26). In both cases branching is possible, so the singular strategy is a branching point at the ESS-branching transition.

Additional references

35. Metz, J. A. J., Nisbet, R. M. & Geritz, S. A. H. The canonical equation of adaptive dynamics for mendelian diploids and haplo-diploids. *Interface Focus* **3**, 20130025 (2013).

36. Geritz, S. A. H., Metz, J. A. J. & Rueffler, C. Mutual invadability near evolutionarily singular strategies for multivariate traits, with special reference to the strongly convergence stable case. *J. Math. Biol.* **72**, 1081–1099 (2016).

37. Ito, H. C. & Dieckmann, U. Evolutionary branching under slow directional evolution. *J. Theor. Biol.* **360**, 290–314 (2014).

38. Govaerts, W. *Numerical Methods for Bifurcations of Dynamical Equilibria* (SIAM, Philadelphia, PA, 2000).

39. Kuznetsov, Yu. A. *Elements of Applied Bifurcation Theory* (Springer-Verlag, Berlin, 2004), 3rd edn.

40. Meijer, H. G. E., Dercole, F. & Oldeman, B. E. Numerical bifurcation analysis. In Meyers, R. A. (ed.) *Encyclopedia of Complexity and System Science*, 6329–6352 (Springer-Verlag, Berlin, 2009).

41. Allgower, E. L. & Georg, K. *Numerical Continuation Methods: An Introduction* (Springer Verlag, 1990).
